# Depression, aging, and immunity: implications for COVID-19 vaccine immunogenicity

**DOI:** 10.1186/s12979-022-00288-7

**Published:** 2022-07-14

**Authors:** Bart N. Ford, Jonathan Savitz

**Affiliations:** 1 Department of Pharmacology & Physiology, Oklahoma State University Center for Health Sciences, Tulsa, OK USA; 2 Laureate Institute for Brain Research, Tulsa, OK USA; 3 Oxley College of Health Sciences, The University of Tulsa, Tulsa, OK USA

**Keywords:** Aging, Inflammaging, Immunosenescence, Psychiatric disorders, Depression, Vaccine immunogenicity, SARS CoV-2

## Abstract

The aging process can have detrimental effects on the immune system rendering the elderly more susceptible to infectious disease and less responsive to vaccination. Major depressive disorder (MDD) has been hypothesized to show characteristics of accelerated biological aging. This raises the possibility that depressed individuals will show some overlap with elderly populations with respect to their immune response to infection and vaccination. Here we provide an umbrella review of this literature in the context of the SARS CoV-2 pandemic. On balance, the available data do indeed suggest that depression is a risk factor for both adverse outcomes following COVID-19 infection and for reduced COVID-19 vaccine immunogenicity. We conclude that MDD (and other major psychiatric disorders) should be recognized as vulnerable populations that receive priority for vaccination along with other at-risk groups.

## Introduction

Immunosenescence is the general term that captures the gradual decline in function of the immune system that occurs during normal aging. Multiple ailments associated with aging can be traced to immune dysfunction including slower wound healing, autoimmune disease, cardiovascular disease, and cancer [1]. Many of these diseases have an inflammatory component. Nevertheless, both pro- and anti-inflammatory biomarkers are elevated in the blood of the old compared to the young in the absence of obvious infection, a phenomenon termed inflammaging [2]. A principal components analysis (PCA) of 19 inflammation-associated biomarkers in 1010 participants between the ages of 21 and 96, identified two axes that explained 29% of the variance [2]. The first axis (PCA1) was comprised of tumor necrosis factor (TNF), soluble TNF receptor I (STNF-RI), STNF-RII, interleukin (IL)-6, IL-18, high sensitivity C-reactive protein (hsCRP), and IL1 receptor A (IL1-RA) and the second (PCA2) was comprised of monocyte chemoattractant protein (MCP), IL-8, and IL-12. PCA1 was strongly associated with age and both principal components were associated with comorbidities and mortality at 6 year follow-up while adjusting for age [2]. Inflammation is a risk factor for cardiovascular diseases, chronic kidney disease, diabetes mellitus, cancer, depression, dementia, and sarcopenia [3]. High levels of IL-6 were associated with frailty in the very old [4]. An inflammatory index that included IL-6 and STNF-RI was a significant predictor of 10-year mortality in 5600 Cardiovascular Health Study participants [5].

The etiology of age-associated inflammation is not fully understood, but various exogenous and endogenous factors are known contributors [6]. Visceral adipose tissue produces excess inflammatory molecules including IL-1β, IL-6, and TNF and contains a high number of resident macrophages [7]. Abdominal body fat typically increases with age, especially in the presence of a poor diet [8]. Diet may also contribute to microbial dysbiosis and “leaky gut” endotoxemia which can activate systemic immune cells [6]. There is also a breakdown of proteostasis, leading to an excess of cellular debris and misfolded proteins in the cytosolic and extracellular spaces [9]. These debris are ligands for certain pattern recognition receptors (PRRs) that initiate inflammatory signaling pathways. Additionally, accumulated cellular or DNA damage from oxidative stress, mitochondrial dysfunction, and telomere attrition can trigger cellular senescence [10]. Senescent cells continue to survive and resist apoptosis, yet the cell cycle is arrested to prevent further proliferation [11]. Although cellular senescence plays an important role in tissue homeostasis, the dramatic accumulation of senescent cells in old age has deleterious effects on damage repair and systemic inflammation [11]. Senescent cells take on a senescence associated secretory phenotype (SASP), in which production of proinflammatory cytokines, chemokines, growth factors, and proteases are increased by selective chromatin alterations [12].

Although baseline levels of inflammatory biomarkers are elevated in the elderly, the cellular response to inflammatory stimuli is diminished. A longitudinal study found a reproducible, persistent reduction in cytokine stimulated T-cell JAK-STAT pathway activation which correlated with baseline CRP concentration [13]. Conversely, dendritic cells (DCs) from elderly subjects secreted more IL-6 and IFNα when stimulated with intracellular human DNA, endotoxin, or ssRNA [14, 15]. However, the DCs have reduced capacity to phagocytose antigen and impaired migration capacity [15]. Likewise, neutrophil phagocytic function and intracellular reactive oxygen production is reduced in the old [16].

The adaptive immune system also undergoes major changes as a result of aging. Bone marrow lymphopoiesis is decreased, reducing the ability to replenish circulating lymphocytes [17]. The thymus is the organ in which bone marrow derived lymphocyte progenitor cells mature into functioning T-cells [18]. The output of a healthy thymus is, in general, a diverse population of non-self-reactive naive T-cells with the ability to recognize and respond to a broad repertoire of foreign peptide antigens presented by MHC complexes. The size and functional space of the thymus shrinks throughout life, reducing the capacity to produce new mature T-cells [19, 20]. Greater thymic atrophy is associated with prolonged stressful events and childhood abuse or neglect [21–23]. Memory T-cells that remain after antigen recognition and activation increase in number so that the total number of T-cells remains relatively constant [24, 25]. In older adults, the architecture of the T-cell compartment consists mostly of memory T-cells and far less of naive T-cells. This renders the adaptive immune system much less prepared to face novel pathogenic infections and is in part responsible for the increased risk of mortality associated with infection in the very old [26–28]. Memory T-cells are also affected by age. Repeated antigenic encounters like those caused by chronic lifelong infectious viruses push the T-cells toward a state of late differentiation [29]. Late differentiated T-cells lose expression of co-stimulatory molecules, such as CD27 and CD28, and may become hyporeactive [29].

Lymph nodes undergo important age-related changes that can diminish the response of the adaptive immune system including an overall decrease in the number of lymph nodes [30]. Healthy lymph nodes have tightly controlled zones where T-cells, B-cells, and antigen presenting cells interact to survey antigenic signals and either mount an active effector response leading to immune memory or induce tolerance to self or non-harmful antigens [31, 32]. Old lymph nodes have less well-defined organizational structure and exhibit fibrosis and adiposity [30]. Overall there is a delayed and diminished lymphocyte proliferation and reduced rate of lymph flow in older lymph nodes [30].

These age-associated changes in adaptive immunity may explain why the very old are at high risk for infectious illness, in particular respiratory viruses. For instance, it is estimated that people over 65 years of age account for approximately 67% of influenza deaths worldwide [33]. Compared to young adults 18–29 years of age, adults aged 65–74, 75–84, and 85 or older are 65, 140, and 340 times more likely to die of COVID-19, respectively [34]. The ability to control latent infections is also diminished with age. For instance, humoral and cellular immunity against latent varicella zoster virus (VZV) from childhood infection (chicken pox) wanes and the elderly become at risk for developing herpes zoster (HZ, shingles) [35].

Adaptive immune decline is also evident in the reduction of vaccine immunogenicity in older recipients. This represents a major challenge in stopping the spread of illness and preventing death in this population. For instance, influenza vaccines are 30 to 50% effective in preventing illness in the elderly compared to 65 to 80% in younger adults [36]. Following influenza vaccination CD8^+^ effector cells from adults over 60 had diminished granzyme B and perforin production and lower cytolytic activity against A/H3N2 compared to younger adults [37]. Influenza vaccine neutralizing antibody response is negatively correlated with the accumulation of late differentiated CD8^+^ CD28^−^ T-cells and serum concentrations of IL-6 in the elderly [38, 39]. Recently, efficacy in the elderly has been somewhat improved by the use of high dose or adjuvanted influenza vaccines [40–42]. Other vaccines are also impacted by immunosenescence. Low memory B-cell frequencies, and high frequencies of pro-inflammatory innate immune cells predicted worse response to hepatitis B vaccination in older adults [43]. Similarly, age-associated CD21^−^CD11c^+^ B-cell frequencies correlated negatively with cellular immune response to HZ vaccine (Zostavax, Merck Sharp and Dohme) [44]. In the section below, we review the effects of aging on the COVID-19 vaccine response.

In this manuscript we generally use the term “vaccine efficacy” to refer to the percentage reduction in disease risk among vaccinated persons relative to unvaccinated persons and we use the term “vaccine immunogenicity” to refer to the strength of the immune response elicited by the vaccine and the durability of this immune response. Nevertheless, the picture is complicated by the fact that the endpoint of COVID-19 vaccine efficacy studies can be variable, being defined variously as protection against severe disease/death, protection against disease, protection against infection, and protection against transmission [45].

## SARS-CoV-2 vaccine response in the elderly

The results from the phase III clinical trials of SARS-CoV-2 vaccines generally did not demonstrate a significant age-related decrement in vaccine efficacy (Table 1). The overall efficacy (defined as prevention of laboratory-confirmed COVID-19 infection) of the BNT162b2 (BioNTech/Pfizer) vaccine (two doses, 3-week interval) against symptomatic SARS-CoV-2 infection was 95.0% and did not differ significantly across individuals aged 16–55 years, > 55 years, > 65 years or > 75 years [46]. Overall efficacy (defined as prevention of symptomatic infection) of the mRNA-1273 (Moderna) vaccine was 94.1% but there was some effect of age with an efficacy of 95.6% for participants < 65 years and 86.4% for participants > 65 years [47]. In contrast, the ChAdOx1 (AstraZeneca/Oxford) vaccine achieved an overall efficacy (defined as protection against symptomatic infection) of 74%. Efficacy was 72.8% in participants < 65 years of age but 83.5% in the > 65 age group [49]. Similarly, the Ad26.COV2.S (Janssen) single-dose vaccine’s overall efficacy against moderate to severe–critical disease was 67% but was higher in individuals > 60 years (76.3%) versus < 60 years of age (63.7%) [50].

**Table 1:** Efficacy of SARS CoV-2 Vaccines by Age Group in Phase III Clinical Trials

Vaccine	Type	Doses (interval)	Median Follow-up	Age Groups	N (vaccine vs placebo)	Efficacy (95% CI)	Primary Efficacy Outcome	Ref.
BioNTech/Pfizer BNT162b2	mRNA	2 (3 weeks)	2 months	All 16–55 > 55	18,198 vs 18,325 10,889 vs 10,896 7971 vs 7950	95.0 (90.0–97.9) 95.6 (89.4–98.5) 93.7 (80.6–98.8)	Confirmed CoVID-19 (symptoms in combination with PCR test)	[46]
Moderna mRNA-1273	mRNA	2 (4 weeks)	63 days	All 18–64 > 64	14,134 vs 14,073 10,551 vs 10,521 3583 vs 3552	94.1 (89.3–96.8) 95.6 (90.6–97.9) 86.4 (61.4–95.2)	Symptomatic Covid-19 with onset at least 14 days after the second injection	[47]
Janssen Ad26.COV2-S	Viral vector	1	58 days	All 18–59 > 59	21,895 vs 21,888 14,564 vs 14,547 7331 vs 7341	66.9 (59.0–73.4) 63.7 (53.9–71.6) 76.3 (61.6–89.0)	Moderate to severe–critical Covid-19	[48]
AstraZeneca/Oxford ChAdOx1	Viral vector	2 (4–12 weeks)	61 days	All 18–64 > 64	21,587 vs 10.792 16,760 vs 8381 4827 vs 2411	74.0 (65.3–80.5) 72.8 (63.4–79.9) 83.5 (54.2–94.1)	Symptomatic illness	[49]

Nevertheless, it should be noted that the older groups made up a relatively small proportion of participants in the phase III trials and therefore there may not have been enough statistical power to detect an age effect. In an Israeli cohort of more than 1 million people, vaccine efficacy (defined as protection against laboratory-confirmed infection) plateaued at 35 days after the second dose of the BNT162b2 vaccine and declined somewhat with age especially in males with an OR for vaccine efficacy of 0.74 in the 81–90-year age group relative to the reference age group [51]. Similarly, in a study of antibody responses in over 200,000 individuals in the UK, recipients of two doses of the BNT162b2 vaccine, showed an antibody positivity rate of > 90% at all ages except 75 years and older, where it was 86.5% [52]. Antibody positivity in recipients of the ChAdOx1 vaccine was over 90% in the younger age groups but dropped to 72% in individuals older than 75 years [52]. In a study of more than 8 million New Yorkers, modest declines in vaccine efficacy (defined as protection against laboratory-confirmed infection) over time were observed in individuals older than 65 years who received the BNT162b2 and mRNA-1273 vaccines [53].

Other smaller studies have focused specifically on vaccine immunogenicity in geriatric samples. In a study of BNT162b2 immunogenicity in the elderly, lower neutralization potency and reduced somatic hypermutation of class-switched cells was observed in individuals greater than 80 years of age [54]. Consistent with these data, Muller et al. reported that individuals older than 80 years of age had approximately 2.8-fold lower antibody titers after two doses of BNT162b2 compared to individuals younger than 60 years of age [55]. Further, after the second vaccination, 2% of the young group versus 31% of the elderly group had no detectable neutralizing antibodies [55]. Similarly, significantly reduced neutralization antibody titers after two doses of the BNT162b2 vaccine were seen in individuals with a median age of 81 years relative to a comparison group of health-care workers with a median age of 34 years [56]. In a comparison of nursing home residents (median age 76) and healthcare worker controls (median age 48), 1.4% of the younger group versus 19% of the elderly group had neutralizing titers at or below the lower limit of detection 2 weeks after the second dose of the BNT162b2 vaccine [57]. Similarly, another study of nursing home residents with a mean age of 83 years reported that 20% of participants failed to show antibody-mediated protective immunity when tested 3–5 months after two doses of BNT162b2 or mRNA-1273 [58]. Nevertheless, neutralization antibody titers were significantly higher in individuals who received mRNA-1273 compared with BNT162b2. Even more striking results were reported in a comparison of 129 adults with a mean age of 44 years and 105 elderly individuals in a long-term care facility with a mean age of 86 years. Two months after the second dose of the BNT162b2 vaccine, neutralizing antibody titers were 10 times lower in the elderly group [59]. These data are consistent with an 8.2-fold reduction in antibody titers reported in recipients of BNT162b2 or ChAdOx1 vaccines in residents of a long-term care facility (mean age of 87 years) versus controls [60]. Nevertheless, the effects of medical comorbidity can be difficult to separate from the effects of age per se, especially when participants are drawn from long-term care facilities.

There are likely to be interactions between age and comorbid medical factors which would not be detected in clinical trials which excluded individuals with immunosuppressive conditions. For instance, in the CAPTURE study which evaluated the efficacy of the BNT162b2 and ChAdOx1 vaccines in cancer patients, older age was associated with a reduced neutralizing antibody response against all SARS-CoV-2 variants tested [61]. In the CANVAX study which evaluated the immunogenicity of BNT162b2, mRNA-1273, and Ad26.COV2.S vaccines in cancer patients, age was negatively associated with the breadth of the neutralization antibody response against SARS-CoV-2 variants [62]. Other studies reported that age greater than 65 years was associated with lack of seroconversion after BNT162b2 or mRNA-1273 vaccines in patients with chronic lymphocytic leukemia [63, 64]. Significant negative effects of age on IgG antibody response to the BNT162b2 vaccine have also been reported in the context of solid organ transplant recipients [65–68], dialysis [69–74], and a spectrum of immunocompromising conditions [75, 76]. Similar reductions in vaccine immunogenicity with advancing age were seen with the Ad26.COV2.S vaccine in patients on dialysis [70] while older patients with inflammatory bowel disease treated with immunosuppressive medications showed a blunted antibody response to BNT162b2, mRNA-1273, ChAdOx1, and Ad26.COV2.S vaccines [77–79].

Third, the median duration of follow-up in the phase III clinical trials was approximately 60 days which may not have been enough to detect differential loss of antibodies over time. Indeed, the reports showing a longer duration follow-up of the phase III clinical trials are indicative of a greater loss of vaccine efficacy in the older age groups. In a small-scale follow-up sample vaccinated with BNT162b2 with a median age of 34 years, antibody responses were negatively correlated with age at 6 weeks (*r* = − 0.19), 12 weeks (*r* = − 0.25), and 6 months (*r* = − 0.29) after the second dose [80]. With respect to the mRNA-1273 vaccine, anti-spike IgG antibodies titers were approximately 40% lower in participants greater than 55 years of age at 90 days after the second vaccination [81]. Similarly, in the 180-day follow-up, geometric mean end-point titers were 92,451 in the 18–55-year-old group, 62,424 in the 56–70-year group, and 49,373 in those 71 years or older [82]. Comparable results were also obtained in a 6-month follow-up of a small cohort of healthy volunteers who received mRNA-1273, with anti-spike titers reduced by approximately two-fold in participants older than 55 years [83]. In the case of the Ad26.COV2.S vaccine, efficacy, defined as protection against moderate to severe-critical disease, was 56.6% in 18–59 year group and 55% in the > 59 year group when Covid-19 onset was least 14 days after vaccination but 54.3 and 46.6% in the younger and older groups, respectively when disease onset was at least 28 days after vaccination [48].

Fourth, the effects of age on SARS-CoV-2 vaccine efficacy are visible in the phase I and II trials and other studies that have examined antibody response after just one dose. For instance, after a single BNT162b2 dose, antibody positivity ranged from 91.5% in the 18–29-year age group to 37.6% in the 70–79-year age group [52]. After a single dose of the ChAdOx1 vaccine seropositivity was 64.9% in the 18–29-year age group compared to 25.0% in 70–79-year-olds [52]. Another study reported that individuals aged 46 years or more were less likely to produce neutralizing antibodies 3 weeks after the first BNT162b2 vaccine dose compared individuals aged 18–45 years [84]. This echoes the phase I study of the mRNA-1273 vaccine, in which anti-spike protein antibody titers were 3 times higher in the 56–71-year age group compared to the > 71 year age group, and two vaccine doses were required to achieve neutralizing antibodies in participants after the age of 56 [85]. In the phase II trial of mRNA-1273, 83% of individuals aged 18–55 years seroconverted after one 100 μg dose whereas in the individuals older than 55 years, the seroconversion rate was 56–78% [86]. Similarly, in an early phase study of the ChAdOx1 vaccine in Japan, neutralizing antibody responses were seen in 67.5, 60.3 and 50.0% of participants aged 18–55, 56–69 and 70 years or more, respectively [87].

Fifth, the clinical trials focused on neutralizing antibody responses since these titers correlate with protection against SARS-CoV-2 in humans [88–91]. T-cell immunity, which is generally measured by interferon-γ (IFNγ) ELISpot or by intracellular cytokine staining using spike antigen peptides, is another important component of vaccine efficacy especially in the case of variants such as Omicron [92]. Collier et al. evaluated T-cell responses by stimulating PBMCs with wild-type SARS-CoV-2 spike protein and using an IFNγ and interleukin-2 (IL-2) FluoroSpot assay to count spike-specific T-cells in participants vaccinated with BNT162b2. Negative correlations between the T-cell responses and age were found (*r* = 0.49) [54]. These data are consistent with a comparison of elderly (median age 81) and younger (median age 34) participants receiving the BNT162b2 vaccine which found that spike-specific T-cell responses were significantly lower in the elderly group [56]. Similarly, in a comparison of elderly (mean age 86) and younger (mean age 44) participants 2 months after the second dose of the BNT162b2 vaccine, spike reactive T-cells were significantly reduced in the older group [59]. Analogous results were reported in a study of 122 lab workers vaccinated with BNT162b2. Approximately 87% of vaccinated individuals developed either CD4^+^ or CD8^+^ T-cell responses at 3 months but CD4^+^ T-cell responses were decreased among vaccinated individuals with elevated levels of senescent CD8^+^ TEMRA cells [80].

Sixth, there is evidence that advancing age has detrimental effects on immunity acquired from natural infection. A Danish study which examined reinfection rates in 2020 (waves 1 and 2), prior to the introduction of SARS-CoV-2 vaccines found that the risk of repeat infection confirmed by PCR was higher among individuals aged 65 years or more compared to the 0–34 year age group with an estimated 47% protection versus 83% protection, respectively [89]. Similarly, in a cohort from the USA, the level of protection against reinfection within 3–9 months after initial infection was approximately 80% in those aged 20–59 years but 67% in those aged 60 years or more [93]. A large Swedish study followed over 30,000 individuals with natural immunity for an average of 164 days and found that after 3 months, a prior infection was associated with a 95% lower risk of reinfection [94]. However, protection was attenuated with age so that the hazard ratio for reinfection was 0.05 in individuals younger than 50 years but 0.55 in those ≥80 years of age. Regarding more severe disease, a small Mexican study reported that increasing age was a risk factor for increased risk of severe symptomatic SARS-CoV-2 reinfection with an RR_per year_ of 1.007 [95]. On the other hand, some studies have reported discrepant results. For instance, an Israeli study performed during the B.1.617.2 (delta) variant wave of the pandemic, examined the waning of natural immunity in a recovered, unvaccinated cohort [96]. After controlling for ethnicity and exposure risk, the number of confirmed infections per 100,000 person-days at risk ranged from 14.2 in 16–39 year-old persons infected 4–6 months previously to 34.9 in individuals infected more than 1 year previously. In the ≥60 year category, the equivalent number of confirmed infections per 100,000 person-days at risk ranged from 4.2 to 12.4 [96].

## Depression may accelerate biological aging

Although certain aspects of immune aging begin early in life, such as thymic involution, the onset and rate of immunosenescence is highly variable and the factors that contribute to the variance are not completely understood [97]. There are many factors that may contribute to immune aging in an individual including genetics, environmental factors, diet, health behaviors, and allostatic load. Here we focus on depression which has been previously proposed to be a driver of premature biological aging [98].

Depression is associated with higher risk of age-related illnesses such as coronary disease, ischemic heart disease, and type 2 diabetes mellitus [99]. Depression often precedes Alzheimer’s disease and Parkinson’s disease, although it is unknown whether this represents a risk factor or early symptom [99]. Depression is also a risk factor for all-cause mortality after controlling for suicidality [100–102]. On a cellular level, major depressive disorder (MDD) has been associated with reduced telomere length, a hallmark of aging with meta-analyses indicating a small to medium effect size but substantial heterogeneity across studies [103, 104]. Epigenetic clocks predict chronological age based on accumulation of methylated CpG sites throughout the genome. Recently, the “GrimAge” clock was developed by incorporating genome methylation and time-to-death data and was found to be a better predictor of mortality than previous epigenetic clocks [105]. In a sample of somatically healthy unmedicated individuals with MDD, the predicted age relative to chronological age was significantly greater than healthy controls while controlling for sex, smoking and body mass index (BMI) [106]. Childhood trauma in pre-menopausal women is also associated with greater GrimAge [107]. RNAseq studies are also suggestive of greater expression of age-associated genes in the peripheral blood mononuclear cells (PBMCs) of MDD participants, potentially indicative of greater biological aging [108]. A composite index of the Senescence-Associated Secretory Phenotype was found to differentiate MDD participants from controls in the NESDA cohort [109]. At the level of brain, an ENIGMA consortium study of several thousand individuals found that people with MDD had a higher predicted brain age compared to controls although the effect size was small, a 1.08-year increase [110]. A recent meta-analysis drew a similar conclusion [111].

Depression is also associated with reduced vaccine immunogenicity and maintenance of immunity. For instance, adults with current or remitted MDD who were born after the introduction of the measles vaccine were less likely to test positive for measles IgG antibodies compared with health controls without psychiatric illness [112]. This could indicate that long term immunity is disrupted by the onset of depression. Similarly, MDD was shown to be associated with lower cellular immunity to VZV at baseline and attenuated responses to live-attenuated HZ vaccination (Varivax, Oka/Merck) in the elderly, indicating that depression can exacerbate the effects of immune aging [113, 114]. Interesting, MDD subjects taking anti-depressive medication had normal responses to vaccination [114]. Also, in a randomized control trial, a relaxation-response intervention, Tai Chi Chih, improved the cell-mediated immune response to VZV compared with a health education control arm [115]. In fact, although the two groups did not differ in VZV responder cell frequency (RCF) at baseline, 16 weeks of Tai Chi Chih prior to vaccination, was associated with as great an increase in VCV-RCF as vaccination was in the control group. Further, vaccination in the Tai Chi Chih arm brought VZV-RCF up to levels observed in a group 30 years younger [115]. These results suggest that depression not only mediates some aspects of immunosenescence but is also an attractive target of therapeutics aimed at improving age-related health outcomes.

The mechanisms underlying this putative acceleration in biological aging are still a matter of debate. Depression is associated with chronic activation of neuroendocrine stress pathways that can alter the immune system and perhaps influence immune aging. For instance, glucocorticoids can contribute to thymic involution, placing a greater reliance on the memory T-cell compartment [116]. Oxidative stress, cortisol, and catecholamine exposure has been linked to the shortening of telomeres [117] with insulin resistance as a possible mediator [118]. Various adverse social conditions have been associated with an up-regulation of inflammatory genes and a down-regulation of type 1 interferon and viral genes, denoted the conserved transcriptional response to adversity [119]. In this model, chronic sympathetic signaling modulates the epigenetic state of myeloid cells in a way that favors proinflammatory NFκB and AP-1 transcription factor activity and inhibits interferon response elements [119]. Thus, this model explains how stress imprints on the immune system, leading to elevated inflammatory activity and suppressed adaptive immunity, similar to immunosenescence.

The suppressed antiviral response in depression may increase susceptibility to immune-altering infections. This factor may be an overlooked source of accelerated immune aging. For instance, we previously found that in two independent samples of depressed adults, childhood trauma was associated with increased odds of seropositivity for cytomegalovirus (CMV) [120]. CMV is a ubiquitous herpesvirus that establishes lifelong latent infections and is considered a driver of immunosenescence [25]. The effort by the immune system to maintain CMV in a latent, subclinical state can lead to T-cell exhaustion and expansion of an oligoclonal CMV-specific memory T-cell population [25]. This can leave the very old at risk for infection by novel pathogens. Indeed, CMV seropositivity is a considered a risk factor for mortality over the age of 80 [121]. Our group found a large effect of CMV on the accumulation on CD27^−^ CD28^−^ revertant effector memory CD4^+^ and CD8^+^ T-cells in an adult sample, but surprisingly this effect was not exacerbated by the presence of MDD [122]. However, in subsequent studies, we found an MDD-specific association of CMV with reduced gray matter volumes, white matter integrity, and resting-state connectivity [123–125]. This could indicate that CMV also influences brain structural and functional changes associated with aging in a way that impacts people with depression to a greater extent. If early life stress increases the likelihood of infection upon exposure, then this may be another important pathway to premature immune aging.

Another possible link between depression and immune aging is sleep. Sleep disturbance is a core diagnostic criterion for MDD, and can take the form of hypersomnia or insomnia. Similarly in elderly adults, night-time sleep duration and subjective sleep quality are reduced [126]. Proper sleep maintains health in part by regulating neuroendocrine and autonomic control of the immune system [127]. With normal sleep, diurnal fluctuations of growth hormones, corticosteroids, and catecholamines maintain immunity by down-regulating inflammatory activity and promoting effector cell surveillance of the periphery during the day [128]. Late in the evening and in early sleep, inflammatory signals rise, drawing the cells into the lymph nodes where memory responses to foreign antigens can be created [128]. Disruptions of sleep are related to suppressed adaptive immune function. For instance, in healthy young adults, chronic and acute sleep deprivation has been associated with poor antibody response to influenza vaccination and with susceptibility to developing a clinical cold following rhinovirus exposure [129–132]. Although depression-associated sleep disruptions may exacerbate immune dysfunction, the relationship with specific markers of immunosenescence are understudied. In some of the few studies addressing this issue, shorter leukocyte telomere length was associated with insomnia and poor sleep quality in younger and older adults [133–137] while in the Women’s Health Initiative study, Carrol and colleagues examined 2078 women with a mean age of 64.5(±7.1) and found that insomnia symptoms were associated with increased epigenetic age and accumulation of late differentiated CD8^+^CD28^−^CD45RA^−^ T-cells [138]. Short sleep was also associated with fewer naïve CD8^+^CD45RA^+^CCR7^+^ T-cells.

Ultimately, it is still unknown whether the immune changes associated with depression represent true advanced immunological aging or if the phenomena are merely qualitatively similar. Long term studies could follow participants before and after anti-depressive therapy to determine if successful treatment attenuates age-related immune decline compared to non-successful treatment. Anti-aging therapeutics such as rapamycin could be tried in depressed subjects to determine if age-related outcomes are altered independently of mental health status. More studies of premature biological aging in the context of MDD are necessary to clarify the complex relationship between these two factors and to identify targets for interventions to attenuate the negative consequences of both aging and depression. In the sections below, we provide an umbrella overview of how depression may alter immune responses to COVID-19 infection and vaccines.

## Depression and other psychiatric disorders as risk factors for COVID-19 disease severity

The literature suggests that pre-existing psychiatric disorders, especially mood and psychotic disorders, increase the risk of morbidity and mortality after SARS-CoV-2 infection. A study of over 2.5 million electronic health records found that people with mood disorders and schizophrenia were more likely to die from COVID-19 infection than the reference group after adjusting for a variety of demographic factors and comorbid conditions with ORs of 2.76 and 3.74, respectively [139]. In a meta-analysis of 23 studies comprising approximately 1.5 million individuals, De Picker and colleagues reported that the presence of any psychiatric disorder doubled the risk of dying from COVID-19 and more than doubled the risk of hospitalization (OR = 2.24) [140]. The mortality effect appeared to be driven by psychotic (OR = 2.05) and mood disorders (OR = 1.99) and did not hold for anxiety disorders (OR = 1.07). Similarly, another meta-analysis of 16 studies reported that mental disorders increased the risk for COVID-19 mortality (crude OR = 1.75; adjusted OR = 1.38) [141]. Again, the effect was driven by severe mental disorders, defined as schizophrenia-spectrum and bipolar disorders, with crude and unadjusted ORs of 2.26 and 1.67, respectively [141]. McIntyre and colleagues focused their meta-analysis of 21 studies specifically on mood disorders [142]. The authors reported an association between pre-existing mood disorders and risk of hospitalization (OR = 1.31) and COVID-19–related death (OR = 1.51) but not increased susceptibility to COVID-19 (OR = 1.27) [142]. A recent systematic review painted an even more concerning picture, estimating a 2–4-fold increased risk of mortality in patients with schizophrenia and 1.5–2-fold greater mortality risk in individuals with MDD [143].

Several potential mechanisms are proposed to explain the link between psychiatric disorders and COVID-19 disease severity. First, several psychiatric disorders, especially mood and stress-related disorders, are characterized by impairments in anti-viral immunity and vaccine immunogenicity [112, 114, 120, 144], raising the possibility of a less effective immune response against SARS CoV-2. These deficits in adapative immunity are consistent with the premature aging hypothesis of depression but extant publications are epidemiological rather than mechanistic in nature. Second, there is a higher rate of cardiovascular, metabolic, inflammatory and other systemic diseases in severe mental illnesses than the general population [145–149], and these diseases are associated with more severe COVID-19 disease [150–152]. Nevertheless, the above meta-analyses still show increased (albeit attenuated) mortality in individuals with psychiatric disorders after adjusting for comorbid medical conditions. Thus, co-occurring medical disorders cannot fully account for the greater severity of COVID-19 disease in people with psychiatric disorders. Third, severe mental illness is often linked with lower socioeconomic status (SES) [153–155], which is in turn associated with reduced access to health care [156, 157]. However, again this factor is unlikely to fully explain the away the findings as population-based studies that did not limit participants to those treated in hospitals, were the focus of at least one meta-analysis [141]. Fourth, biological aging has been hypothesized to be accelerated in multiple psychiatric disorders [110, 158, 159] and older age is a risk factor for adverse outcomes after SARS-CoV-2 infection [160, 161]. Fifth, psychiatric medications have been proposed as potential moderators of COVID-19 disease severity [143], although teasing apart the effects of psychiatric disorders from comorbid medical conditions, and the direct pharmacological effect of various classes of anti-depressants, mood-stabilizers, and anti-psychotic medications, is challenging. Sixth, social and lifestyle factors associated with psychiatric illnesses such as substance use [162, 163], lack of exercise [164], sleep disturbance [131], and social isolation [165] have all been independently linked with impaired viral immunity or susceptibility to COVID-19, more specifically. Opioids in particular, have respiratory depressant effects that could contribute to mortality in the case of COVID-19 [141]. Seventh, people with severe mental illness may be more likely than the general population to be exposed to SARS-CoV-2 because they reside in psychiatric inpatient units, homeless shelters, community housing, or prisons [142]. Eighth, there may be genetic factors (e.g. variation in human leukocyte antigen genes) that may predispose to certain psychiatric disorders and influence the nature of the anti-viral immune response to SARS CoV-2 [141, 166]. Finally, psychiatric disorders, especially MDD, bipolar disorder, and schizophrenia, are associated with elevated concentrations of inflammatory cytokines in the absence of obvious infection, similar to inflammaging [167–171]. Cytokine release syndrome (CRS) is a hyper-activation of the inflammatory response that contributes to severe acute repiratory distress syndrome (ARDS) and organ failure during SARS-COV-2 infection [172, 173]. Although the predictive role of low-grade inflammation prior to infection in COVID-19 outcomes is not known, it stands to reason that individuals with abnormal inflammatory regulation at baseline may be more vulnerable to CRS. One obvious challenge in addressing this possibility is the need for pre-infection measurements of circulating cytokines during a time window likely to reflect the state of the patient during infection. Nevertheless, other comorbidities with an inflammatory component such as obesity and cardiovascualar disease are also predictors of severe COVID-19 [150–152].

While pre-existing severe mental illness is a risk factor for adverse outcomes following COVID-19, the picture is further complicated by the fact that COVID-19 increases the risk of developing incident psychiatric disorders. Most studies and therefore systematic reviews have focused on symptoms as opposed to diagnosed psychiatric disorders per se. For instance, in their systematic review, Badenoch and colleagues found that the most prevalent neuropsychiatric symptom following COVID-19 infection was sleep disturbance (27%) followed by fatigue at 24% [174]. Depression had a lower prevalence at 13% and there did not appear to be a significant effect of hospitalization status, severity, or duration of follow-up [174]. Another systematic review of neuropsychiatric symptoms reported a point prevalence of 23% for depression based on 10 studies [175]. Based on 46 included studies, Schou et al. concluded that COVID-19 is a risk factor for developing depression and anxiety symptoms although these authors noted that symptoms appear to resolve over time [176]. The problem with these data is that they are confounded by the inclusion of studies in which the baseline psychiatric status of participants was not always clear, and thus most cases of COVID-19-associated psychiatric illness were likely preexisting. Arguably the clearest data come from a large retrospective study that used the electronic health medical records of 236,000 people to examine the incidence of neurological and psychiatric outcomes in the 6-month period after a confirmed diagnosis of COVID-19 [177]. The incidence of a new mood disorder was 3.86% in non-hospitalized patients, 4.49% in hospitalized patients, and 5.82% in patients who were treated in intensive care units [177]. For a new episode of psychosis, the incidence ranged from 0.25 to 0.89%. Harrison and colleagues attribute these findings to several potential mechanisms including viral infection of the CNS, hypercoagulable states, and the neural effects of a systemic inflammatory response [177].

## COVID-19 vaccine response and mental health

When assessed 21 days after a second dose of the BNT162b2 vaccine, people with a diagnosis of depression were less likely to be seropositive than the reference group after adjusting for age and sex (OR = 0.74) [52]. In the fully adjusted models which included age, sex, ethnicity, previous COVID-19 infection, vaccination status, adiposity, and smoking status among other variables, the results remained significant with an OR of 0.83 [52]. It is noteworthy that in this cohort the effect size for depression was in the same ballpark as other medical conditions that have attracted more research with respect to COVID-19 vaccine immunogenicity such as cancer (ORs 0.68–0.77), diabetes (ORs 0.71–0.75), or an immunocompromising disorder (ORs 0.52–0.67). On the other hand, there did not appear to be significant effect of anxiety with ORs of 0.93–1.13. For “other psychiatric disorders”, the OR was 0.76 in the model adjusted for age and sex but 1.01 in the fully adjusted model [52]. In contrast, a population based study of individuals who received two doses of ChAdOx1 (*n* = 5770) or BNT162b2 (*n* = 3331) failed to demonstrate a significant effect of depression/anxiety (considered as a single category) on the presence or absence of anti-spike protein antibodies [178]. Another study of over a quarter of a million individuals found that people with any psychiatric disorder were at an increased risk of a breakthrough COVID-19 following vaccination with an OR of 1.03–1.07 depending on the statistical model [179]. For MDD, the relative risk ranged from 1.05–1.11 but for both MDD and other psychiatric disorders, the effect was strongest in the participants aged 65 years or more even after controlling for medical comorbidity [179]. As with COVID-19 pathology, exactly which pre-existing immune phenotypes predict vaccine immunogenicity or efficacy have not been fully investigated. Experimental study designs are needed to identify influencing factors, such as inflammatory and antiviral cytokine concentrations or immune cell phenotypes, that could reveal the mechanisms of depression-associated adaptive immune suppression.

## Conclusions

Depression may lead to accelerated biological aging via several different intersecting molecular pathways. Aging is in turn, a major risk factor for adverse outcomes following COVID-19 and is associated with decreased efficacy of vaccination, including impaired antibody responses to the current SARS CoV-2 vaccines. This may explain the emerging data suggesting that people with depression are at risk for more severe COVID-19 disease and are less likely to be protected from COVID-19 following vaccination, effects that may be further amplified in elderly depressed samples. Nevertheless, there is far less work in this area relative to other medical fields such as cancer, autoimmunity or transplantation despite indications that major psychiatric disorders are a significant source of risk. More research is needed to elucidate the underlying mechanistic pathways. Nevertheless, based on the modest amount of research published to date, we suggest that MDD (and other major psychiatric disorders) should be recognized as vulnerable populations that receive priority for SARS CoV-2 vaccination along with other at-risk groups.

## Data Availability

Data sharing is not applicable to this article as no datasets were generated or analyzed during the current study.
